# EEG Sensor-Based Computational Model for Personality and Neurocognitive Health Analysis Under Social Stress

**DOI:** 10.3390/s25247634

**Published:** 2025-12-16

**Authors:** Majid Riaz, Pedro Guerra, Raffaele Gravina

**Affiliations:** 1 Department of Informatics, Modeling, Electronics and System Engineering, University of Calabria, 87036 Rende, Italy; majid.riaz@dimes.unical.it; 2 Department of Personality, Assessment and Psychological Treatment, University of Granada, 18071 Granada, Spain; pguerra@ugr.es

**Keywords:** biosignal processing, cognitive resilience, electroencephalography (EEG), human-centric AI, machine learning, mental health, neuroscience, neural oscillations, personality traits, social stress, theta–beta ratio (TBR)

## Abstract

This paper introduces an innovative EEG sensor-based computational framework that establishes a pioneering nexus between personality trait quantification and neural dynamics, leveraging biosignal processing of brainwave activity to elucidate their intrinsic influence on cognitive health and oscillatory brain rhythms. By employing electroencephalography (EEG) recordings from 21 participants undergoing the Trier Social Stress Test (TSST), we propose a machine learning (ML)-driven methodology to decode the Big Five personality traits—Extraversion (Ex), Agreeableness (A), Neuroticism (N), Conscientiousness (C), and Openness (O)—using classification algorithms such as support vector machine (SVM) and multilayer perceptron (MLP) applied to 64-electrode EEG sensor data. A novel multiphase neurocognitive analysis across the TSST stages (baseline, mental arithmetic, job interview, and recovery) systematically evaluates the bidirectional relationship between personality traits and stress-induced neural responses. The proposed framework reveals significant negative correlations between frontal–temporal theta–beta ratio (TBR) and self-reported Extraversion, Conscientiousness, and Openness, indicating faster stress recovery and higher cognitive resilience in individuals with elevated trait scores. The binary classification model achieves high accuracy (88.1% Ex, 94.7% A, 84.2% N, 81.5% C, and 93.4% O), surpassing the current benchmarks in personality neuroscience. These findings empirically validate the close alignment between personality constructs and neural oscillatory patterns, highlighting the potential of EEG-based sensing and machine-learning analytics for personalized mental-health monitoring and human-centric AI systems attuned to individual neurocognitive profiles.

## 1. Introduction

Individuals vary widely in their behaviors, preferences, lifestyles, experiences, and social interaction patterns, these differences shaping the foundation of individual personality development [1]. Personality characterizes an individual’s unique way of thinking and responding to daily life challenges, including decision-making, problem-solving, health management, and routines such as eating and sleeping. Personality traits tend to remain stable throughout different stages of life, making them a key focus for psychologists aiming to understand their development and impact. In recent years, researchers across computer science, medicine, and psychology have been very active in studying personality as it is practically applicable in business, marketing, education performance, recommendation systems, and emotional assessment.

**Figure 1:**
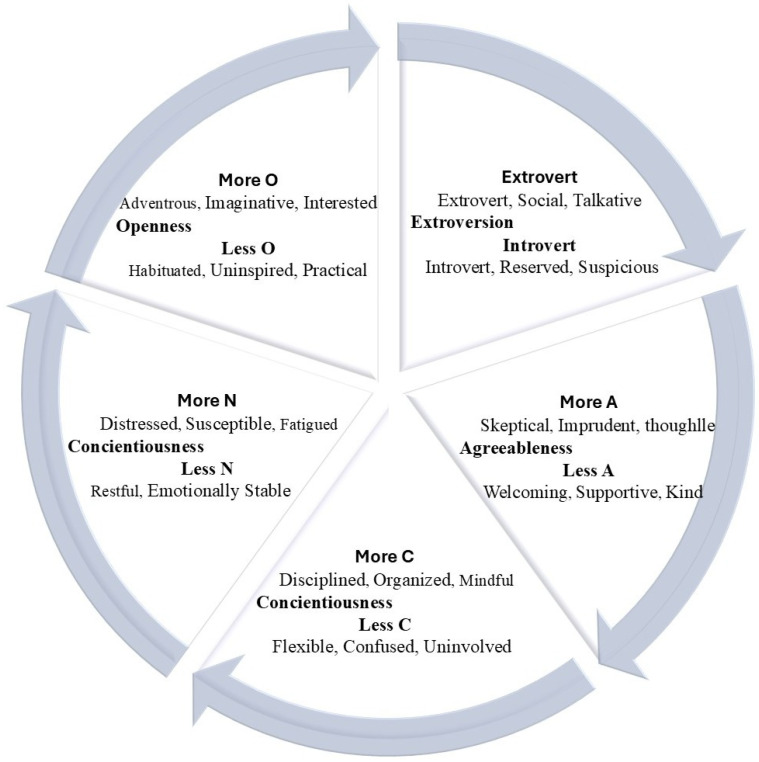
Five-factor model of personality traits with facet explanations.

Numerous theories describe human personality traits based on habits, attitudes, social interactions, and behaviors throughout various situations of daily life. Among these, the five-factor model (FFM), commonly known as the Big Five, is the most widely adopted framework for categorizing human personality into five dimensions: Extraversion, Agreeableness, Conscientiousness, Neuroticism, and Openness to Experience [2]. Each of these dimensions represents a spectrum, with opposing qualities at each end [3], as depicted in Figure 1. Understanding and exploring personality traits is valuable from various perspectives, including self-awareness, natural language processing [4], informed decision-making, career growth, fostering relationships, recommendation systems, improving health, and learning cultural dynamics. In this research, a novel experiment has been conducted using the Trier Social Stress Test (TSST) to identify the five main personality traits from the patterns of brainwaves generated during a set of social stress-related tasks. This study seeks to identify the Big Five personality traits from brainwave patterns evoked during a set of tasks related to social stress. The TSST is widely used for stress induction, negative emotion elicitation, and assessment of working memory [5], as well as to evaluate personal attitudes and individual differences in psychobiological responses [6].

Although various factors have been studied for their effects on cognitive health and memory performance, the role of personality traits and their interaction with physiological responses in maintaining cognitive well-being is not yet understood. For instance, ref. [7] examined the relationship between the five major personality traits and cognitive impairment or dementia, relying solely on statistical analyses. Despite significant advancements in mental health research, stress analysis, and behavioral modeling for enhancing subjective well-being, the intricate mechanisms linking personality traits to cognitive health, particularly in highly stressful environments, remain largely unexplored [8]. This work, therefore, pioneers the investigation of links between personality traits and cognitive health by examining brain responsiveness to psychosocial stress. Specifically, the motivation for this study is detailed as follows:

1. Recognizing EEG-based personality traits in response to the Trier Social Stress Test: This study aims to quantify how brain electrical pulses reflects personality traits, particularly under psychosocial stress. With advancements in wearable EEG technology, monitoring individual differences in real-world contexts has become increasingly accessible.
2. Assessing personality-driven neurocognitive markers of health: In today’s high-stress environments, understanding how personality traits influence mental and cognitive health is crucial. Evidence shows that stress severely impacts mental health, with significant variability at individual levels. Tailored approaches are therefore necessary to support individual cognitive well-being.
3. Understanding personality, cognitive health, and stress interactions: Personality traits critically influence how individuals adapt to social stressors, yet the neurocognitive mechanisms linking these traits to health outcomes remain unclear. The current approaches often isolate psychological or neural factors, neglecting their integration during dynamic social interactions. To address this gap, we develop an EEG-based computational model to identify personality-specific neural markers under social stress. By analyzing EEG data during ecologically valid stress tasks, we decode how traits predict resilience or vulnerability in real-time neural dynamics. These markers offer novel biomarkers for early detection of mental health risks and pave the way for personalized interventions tailored to individual neurocognitive profiles. This work bridges personality psychology and computational neuroscience, advancing precision frameworks to map brain–behavior interactions in socially stressful contexts.

### 1.1. Objectives

The main objectives of the study are as follows:

- To examine the potential of EEG brainwave patterns in identifying the Big Five personality traits in response to psychosocial stress induced by the TSST.
- To evaluate the influence of different personality traits on neural rhythms across various high-stress conditions.
- To explore the relationship between personality traits and cognitive health through EEG-based bioindicators by investigating how different personality traits influence or moderate cognitive performance in sustained stress environments.

### 1.2. Contributions

We developed an entirely new EEG dataset specifically for personality trait recognition, collected from 21 subjects using a 64-electrode EEG headcap. To obtain ground truth data for personality assessment, the participants completed Big Five personality questionnaires. Stress induction was systematically achieved through the TSST, divided into four distinct sessions. After each psychosocial stress session, neural responses across different personality dimensions were evaluated to identify which specific traits are more vulnerable to stress and which recover more rapidly. This dimension-specific analysis contributes uniquely to the field by informing the development of personalized healthcare approaches that are tailored to individual stress resilience and recovery profiles.

Additionally, this study addresses notable gaps in personality assessment by evaluating personality traits as moderators of neurocognitive health. We present a groundbreaking computational framework leveraging EEG-derived neural activity captured during ecologically valid stress paradigms to (1) quantitatively map individual personality traits and (2) establish predictive correlations between these traits and domain-specific neurocognitive health markers. This first-of-its-kind model bridges computational neuroscience and psychometric profiling, offering a transformative lens to decode brain–behavior–personality interactions in dynamic social contexts. For identifying personality characteristics from brainwave spectral bands, two supervised machine learning approaches—support vector machine (SVM) and multilayer perceptron (MLP)—were employed. To delve deeper into the dynamic relationship between personality and cognitive health, the theta–beta ratios (TBRs) from the frontal and temporal brain regions across all the EEG electrodes were assessed as key bioindicators [9]. Variations in brain activity were observed for the personality groups across each phase of the TSST, and the influence of personality traits on cognitive health was further examined by correlating personality scores with TBRs across all the TSST phases.

While our exploratory study utilized a cohort of 21 participants to address pilot objectives, we implemented rigorous statistical methodologies to ensure robust and unbiased inference. This included (1) intra-individual correlation analyses to evaluate interdependencies among the Big Five personality traits and (2) phase-specific correlations between personality traits and frontal/temporal TBRs across all the TSST stages. These multifaceted analytical frameworks—designed to mitigate population limitations—exceed the methodological scope of comparable state-of-the-art studies with analogous sample sizes, which often lack external validation or granular physiological–behavioral integration. We acknowledge generalizability constraints inherent to exploratory cohorts but emphasize that our approach aligns with, and expands upon, the established norms in hypothesis-generating neuroaffective research. Future large-scale validation will further contextualize these findings.

The remainder of the paper is organized as follows: Section 2 presents a review of the literature, where we explore various cutting-edge techniques. Section 3 covers the methodology, Section 4 presents the detailed experimental results, Section 5 provides the discussion, and Section 6 offers conclusions and suggestions for future work.

## 2. Literature Review

Various subjective and objective methods have been used to assess personality traits across different real-life situations. The subjective methods primarily involve questionnaires, while the objective approaches encompass psychophysiological techniques, where physiological signals are collected from participants using wearable and non-wearable sensors. Recently, wearable sensors have shown great value in offering critical insights into human physiology to assess personal behaviors and personality traits that impact quality of life and the aging process [10]. In the past few years, numerous studies have focused on identifying personality traits through various physiological signals, such as electroencephalography (EEG), galvanic skin response (GSR), photoplethysmogram (PPG), electrocardiogram (ECG), electromyogram (EMG), eye-tracking, and facial expressions. For instance, in [11], EEG, GSR, and PPG were simultaneously recorded during public speaking to assess personality traits. Studies such as [12,13,14] identified personality dimensions using EEG data. The dynamic interaction between personality traits and online teaching has been examined using ECG, GSR, and facial EMG [15]. Similarly, in [16], affective video clips were shown to classify personality traits based on EEG, ECG, GSR, and facial expressions. In [17], it was shown that personality traits can be identified using visual and prosodic features. Recent studies demonstrate that deep learning architectures—such as spatio-temporal representation learning fusion networks and convolution-based capsule networks—can outperform traditional machine learning methods in emotion and personality recognition [18,19]. These models leverage hierarchical feature extraction and temporal–spatial encoding to capture complex neural dynamics more effectively than classical approaches. However, for the present study, we intentionally relied on conventional machine learning models to maintain interpretability, reduce computational complexity, and ensure methodological robustness given the limited dataset size. Future work will explore the integration of deep learning frameworks once a larger dataset becomes available, enabling a more comprehensive evaluation of their potential advantages in EEG-based personality and neurocognitive modeling.

In addition to using implicit data for recognizing personality traits, explicit methods have been employed to identify personality dimensions. Researchers have developed significant tools, such as the Big Five Inventory (BFI) [20] and the International Personality Item Pool (IPIP) [21], which are widely used and publicly accessible. Since personality is an implicit aspect of an individual and cannot be fully assessed through questionnaires alone, psychologists have also developed alternative methods for identifying specific personality types. Large Language Models (LLMs) can infer personality traits from digital footprints on social media [22], such as Facebook profile photos, Instagram posts, and personality assessments based on Twitter activity [23]. This AI-driven approach leverages status updates, music playlists, comments, browsing history, and likes and dislikes along with machine learning techniques to predict real-life behaviors, habits, personal values, attitudes, and overall personality traits.

In recent years, human mental health has gained paramount importance as maintaining cognitive well-being has become a priority amid an increasingly competitive environment and the rapid advancement of smart technologies. Cognitive workload essentially measures the mental effort or complex multifaceted demands needed for individuals to accomplish tasks effectively [24]. A vast range of physiological metrics have been utilized to assess cognitive load within real-world environments. For instance, ref. [25] distinguished between cognitive workload and stress levels in office settings using electrodermal activity (EDA)-derived features, while ref. [26] explored machine learning techniques to classify cognitive workload via EEG signals. Studies have shown that EEG, ECG, and GSR measures effectively identify and analyze mental stress across various workplaces [27]. Similarly, ref. [28] utilized EEG, ECG, and eye-tracking to classify subjective cognitive decline in drivers. Additionally, distinct cognitive states—such as distraction detected through EEG [29], the identification of neurodegenerative conditions, including dementia, Alzheimer’s disease, and mild cognitive impairment, using biomedical data [30], and cognitive load assessed through cardiovascular metrics [31]—have been quantified using statistical and machine learning methods. Extensive research has demonstrated the use of EEG for predicting epileptic seizures and other neurological conditions [32,33,34].

There is a growing need for early cognitive health markers. Power spectral density (PSD)-based ratios, such as theta/beta (TBR), have emerged as promising indicators. TBR has shown potential in detecting mild cognitive impairment (MCI) in Alzheimer’s disease [9] and moderating the impact of anxiety on attention control [35]. Stress-related studies reveal that alpha/beta and theta/beta ratios decline under pressure [36], while an increase in beta/alpha ratio reflects elevated cognitive load [37,38]. These EEG-based ratios reflect cortical–subcortical dynamics and have been associated with stress, anxiety, and depression. Notably, reduced TBR in the right frontal region correlates with greater depressive symptoms [39], alongside alterations in PSD, power ratios, and phase-locking values [40].

Cognitive health, which represents an individual’s mental functioning, is shaped by multiple factors, such as personal attributes, sociodemographic and lifestyle elements, medical and psychological conditions, task demands, and sociocultural influences [41]. It has been observed that even individuals of the same age, and within the same family, often experience diverse age-related conditions, including cardiovascular and neurodegenerative diseases, along with physical and cognitive declines. Research has identified the key factors underlying these health disparities, explaining why some individuals enjoy good health while others are more susceptible to illness. Among these factors, personality traits have proven essential in influencing the trajectory of healthy aging. Studies have linked personality to health outcomes; for instance, ref. [42] reported that individuals with higher personality scores tend to have greater longevity. The association between biomarkers of active healthy aging and personality traits was examined in [43], while ref. [44] suggested that personality traits may moderate anxiety, particularly in the context of correlations between left and right frontal EEG TBR and anxiety levels. Research on personality inference from EEG signals in response to emotional videos was conducted in [3,12,16], and functional brain mapping studies, such as [45], have assessed Extraversion and introversion, revealing both traits’ alignment with brain health. Multimodal physiological signals—like EEG, ECG, and GSR—have demonstrated effectiveness in personality, affect, and mood analysis [46]. Thus, personality traits present inherent health tendencies that must be comprehensively assessed from both psychological and physiological perspectives to develop a personality-aware healthcare system.

Although many advanced studies have emphasized the importance of cognitive health, the key factors influencing it—such as lifestyle, physical activity, diet, and social engagement—have yet to be thoroughly explored [47]. This paper introduces a unique approach to recognizing personality traits through biological indicators and verified data, examining their influence on cognitive health. Although stress and cognitive workload have been analyzed in various contexts, the specific interplay between cognitive load and personality traits during the TSST via EEG signals is still unexplored. Previous research, such as [37,48], used electrodermal activity (EDA) to classify acute stress states triggered by the TSST, while the TSST has also served to distinguish psychosocial stress through body movement and posture analysis [49]. Another study [50] leveraged the TSST for cognitive performance evaluation using heart rate variability (HRV) features from ECG data. In [37], the Stroop Color–Word Test (SCWT) was utilized to induce and categorize stress.

### Comparison with State-of-Art EEG-Based Personality Recognition

To strengthen and clarify the contextual positioning of our proposed EEG-based personality identification framework, we systematically compared it with several existing approaches, including ASCERTAIN-based studies, emotion-evoked EEG paradigms, low-electrode consumer EEG systems, and short-duration recording protocols. While these studies have contributed valuable insights, they are limited by constrained electrode coverage, brief and non-ecological tasks, or the absence of stress-modulated neural dynamics. In contrast, our work introduces a novel contribution by integrating the Trier Social Stress Test (TSST), a multiphase and ecologically valid stress-induction protocol that evokes robust and dynamic changes in brain activity. This allows us to assess personality traits across stress, baseline, and recovery phases, providing richer and more resilient neural markers for personality inference. Moreover, our study uniquely couples personality-specific EEG patterns with neurocognitive health biomarkers—particularly TBR—an aspect that has not been explored in prior EEG-based personality research. This dual-layer analysis positions our framework as a significant advancement beyond the existing methodologies. Table 1 presents a comparison of state-of-the-art approaches for personality trait recognition using physiological signals. Most existing studies suffer from limited experimental rigor, often relying on affective video stimuli without structured protocols. Their analyses are typically confined to a few brain regions, offering an incomplete view of the neural responses related to personality. Moreover, these studies largely overlook the role of personality traits as mediators of cognitive health under dynamic and stressful conditions, with no consideration for baseline or recovery phases. Further, the connection between personality and neurocognitive health remains underexplored. In contrast, our study addresses these gaps by identifying distinct personality-driven neural patterns across stress phases and by examining how personality traits influence coping mechanisms and align with neurocognitive health indicators. As in previous studies, personality has never been decoded from the brain during social stress conditions, and neurocognitive health through TBR has never been used in previous studies, which is why we comprehensively demonstrate how personality matters in regulating brain health in different circumstances.

## 3. Proposed Methodology

**Figure 2:**
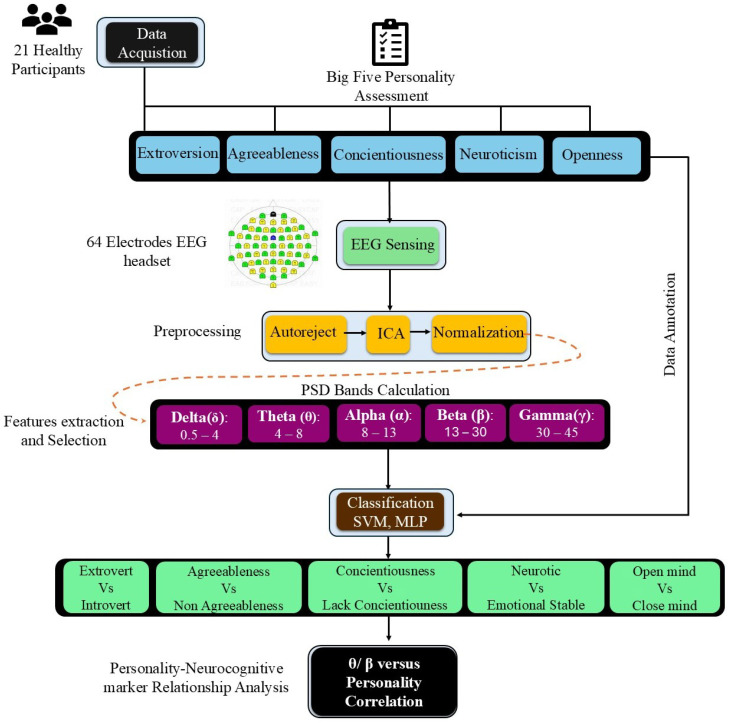
Framework for personality recognition and brain dynamics during TSST.

Figure 2 shows the analysis workflow followed for personality trait recognition, including the data acquisition, feature extraction and selection, classification stages, and cognitive health analysis for personality inference.

While our framework builds on well-established EEG-based analysis methods—incorporating band-power feature extraction, wrapper-based feature selection, and conventional classifiers such as SVM/MLP—its novelty lies in the systematic organization and integration of these components to assess personality traits under different experimental conditions within the context of our acquired dataset. Unlike previous EEG-based personality assessment pipelines, our study evaluates personality across multiple socially relevant conditions, thereby ensuring a diverse dataset and emphasizing interpretability and computational efficiency compared to black-box approaches such as deep representation learning. Furthermore, our study provides empirical evidence linking personality-related patterns to neurocognitive indicators.

### 3.1. Data Acquisition

#### 3.1.1. Participants

A total of 21 doctoral students, aged between 26 and 32, were initially recruited for this study, each voluntarily participating. However, data from two participants were excluded from the final analysis due to excessive noise and artifacts, resulting in a final sample size of 19 participants (9 male, 10 female). All participants reported no significant medical history and were free of any serious illnesses, including neurological disturbances and personality disorders. Each participant was right-handed and had normal or corrected-to-normal vision. Ethical approval for the study was obtained, and informed consent was secured from all participants prior to the experiment.

#### 3.1.2. Experimental Material

For continuous EEG recordings, the BioSemi ActiveView system was used, with data digitized at a sampling rate of 1024 Hz and 24-bit analog-to-digital (A/D) conversion. A total of 64 Ag/AgCl active scalp channels were arranged according to the international 10-20 system standard [51]. Electrodes were placed on the scalp using a nylon cap, with cap size adjusted to fit each participant’s head. Common Mode Sense (CMS) and Driven Right Leg (DRL) served as the ground, with all electrodes using CMS as the reference during signal recording. To ensure optimal electrode contact and signal transmission, Sigma Electro Gel was applied at each electrode position on the cap. Participants were instructed to minimize eye blinks, avoid body movements, and maintain a steady distance from the screen while performing the tasks.

#### 3.1.3. Experimental Protocol

**Figure 3:**
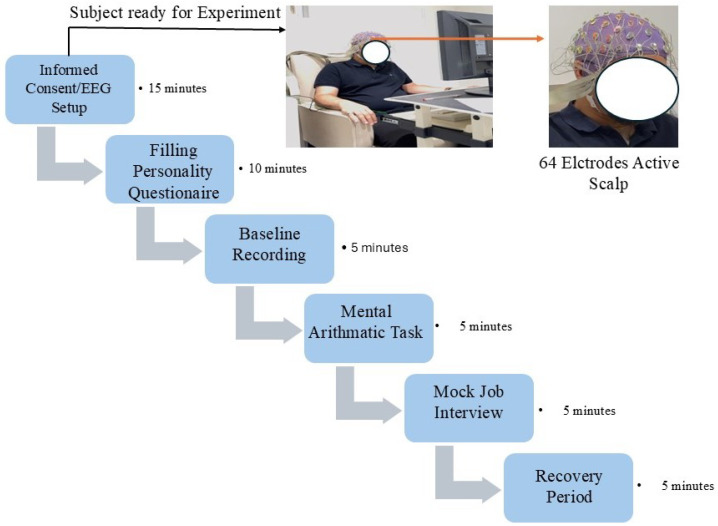
Experimental procedure used for recognizing personality traits during the Trier Social Stress Test.

Participants sat in a quiet room and were briefed on the study aim and procedure, as outlined on the consent form. They first completed an online demographic form on an iPad, followed by the Big Five personality questionnaire (50 questions, 0–5 scale). EEG recording began with a 5-min baseline before starting the TSST. Next, a mental arithmetic task required subjects to count backward from 1022 by 13, restarting on errors with a visible countdown and dynamic scoring adjustments. Afterward, participants prepared for a 5-min mock job interview to maintain stress levels. Finally, the session ended with a recovery phase in which the subjects listened to relaxing music. The entire procedure lasted around 45 min per trial. The experimental procedure is illustrated in Figure 3.

### 3.2. EEG Preprocessing

To enhance EEG signal quality, we applied preprocessing steps to reduce noise and artifacts. First, a 1–45 Hz bandpass filter was used to attenuate unwanted frequencies. We then employed the MNE-Python (version 1.6.1) library, which provides robust algorithms for artifact removal in EEG data preprocessing [52]. Specifically, we applied the AutoReject and Independent Component Analysis (ICA) methods. AutoReject, an automated technique, segmented data into 2-second epochs, calculated optimal thresholds for each channel, and marked noisy segments on a trial basis for removal or interpolation. This step retained cleaner epochs while tracking noisy ones. Following automatic artifact rejection, ICA was applied to further isolate and exclude components associated with residual artifacts, using AutoReject’s identified components for exclusion. This combined approach improved data quality by maximizing artifact reduction while preserving the integrity of the original signal for further analysis.

Our artifact rejection pipeline implemented a rigorous data-driven optimization framework through AutoReject’s adaptive parameter selection. For each experimental session, the algorithm systematically evaluated interpolation strategies across six consensus thresholds (0.10–0.70) and four channel interpolation ranges (1–16 sensors/epoch) using 10-fold cross-validation. This machine learning approach identified optimal parameters by minimizing out-of-sample reconstruction errors, yielding a median consensus threshold of 0.30 (IQR: 0.20–0.50) with conservative sensor interpolation limits of 1–4 channels per epoch. Post-optimization epoch rejection rates exhibited substantial inter-individual variability (0–26.7% per session; 0–40 epochs/150), reflecting task-dependent neurophysiological dynamics. Complementing this, our ICA protocol applied stringent component exclusion criteria, systematically removing artifactual sources contributing <2% variance per decomposition, a statistically conservative threshold ensuring preservation of neurobiologically meaningful signals while eliminating transient artifacts. An overview of the EEG preprocessing workflow for enhancing signal quality and reducing artifacts is presented below.

- **Signal filtration**
  - Bandpass filter (1–45 Hz).
- **AutoReject algorithm**
  - Systematic interpolation.
  - Six consensus thresholds (0.10–0.70).
  - 1–16 sensors/epoch.
  - Post-optimization epoch rejection rates (0–40 epochs/150).
- **ICA**
  - Independent Component Analysis.
  - Removal of artifactual sources contributing <2% variance per decomposition.
- **Z-score normalization and power spectral analysis**
  - Power spectral analysis.
  - Standardization of each subject’s data by subtracting the mean and dividing by the standard deviation across conditions.

During EEG acquisition, all participants remained seated comfortably throughout the entire experiment, and no physical activity was involved. This minimized large muscle movements, and no visible EMG-related artifacts were observed in the raw recordings. These recordings were visually inspected in real time during data collection, and no prominent muscular contamination was detected—consistent with the absence of EMG components removed by ICA or AutoReject. However, we acknowledge that, during the cognitively demanding phases of the TSST, such as the mental arithmetic and job interview tasks, subtle facial and cranial muscle tension may still occur. The prior literature indicates that such low-amplitude low-frequency EMG activity can partially overlap with neural oscillations in the frontal and temporal regions, potentially influencing theta and beta spectral power during stress. Although our visual inspection suggested minimal EMG influence in this dataset, we recognize that ICA alone may not fully eliminate stress-induced EMG components. Future studies will therefore incorporate EMG-specific component identification procedures, additional reference channels, and advanced preprocessing pipelines designed to differentiate overlapping EEG–EMG activity. These methodological improvements will ensure a more rigorous dissociation between neural and muscular sources when examining stress-related EEG dynamics.

### 3.3. Feature Extraction and Normalization

#### 3.3.1. Welch Method of PSD Estimation

To analyze brain activity in the frequency domain, we computed the power spectral density (PSD) from preprocessed EEG data using the Welch method with Fast Fourier Transform (FFT), a highly effective and widely recognized approach detailed in [53], which we found to be particularly suitable and reliable for our analysis. Each EEG signal was divided into segments of 1024 samples with overlap of 50%, providing a robust frequency representation throughout the electrode array. PSD values were integrated over five key frequency bands—delta (1–4 Hz), theta (4–8 Hz), alpha (8–12 Hz), beta (12–30 Hz), and gamma (30–45 Hz)—to capture distinct neural dynamics.

The feature matrix dimension was set to 76 by 320, accounting for 19 subjects, 4 experimental conditions, 5 frequency bands, and 64 electrodes: CP5, CP3, CP1, P1, P3, P5, P7, P9, PO7, PO3, O1, Iz, Oz, POz, Pz, CPz, Fpz, Fp2, AF8, AF4, AFz, Fz, F2, F4, F6, F8, FT8, FC6, FC4, FCz, Cz, C2, C4, C6, T8, TP8, CP6, CP4, P2, P4, P6, P8, P10, PO8, PO4, and O2. The detailed PSD computation across these electrodes is defined in Equation (1).

(1) $$P_{xx}\left(f\right)=\frac{1}{L}\sum_{k=1}^{L}\frac{1}{N}{\left|F\left(w_{k}\left(t\right)\right)\right|}^{2}$$

where $P_{xx}\left(f\right)$ is the estimated power spectral density at frequency *f*, *L* is the number of segments, and $w_{k}\left(t\right)$ is the windowed signal for the *k*-th segment.

#### 3.3.2. Z-Score Normalization

To ensure consistent feature contribution and enable cross-task comparisons, Z-score normalization (also known as standardization) was applied to each mean band power. This transformation subtracts the mean from each value and divides by the standard deviation within each subject’s data, yielding standardized values across conditions. The mathematical formulation of this normalization process is shown in Equation (2).

(2) $$X_{\mathrm{normalized}}=\frac{X-\mu }{\sigma }$$

where

- *X* is the original value;
- μ is the mean of the signal;
- σ is the standard deviation of the signal.

EEG features were extracted using canonical frequency bands supported by the existing literature. Although individualized spectral band boundaries were computed within subjects, the theta–beta ratio (TBR) was analyzed at the group level by contrasting high- versus low-trait participant cohorts. While estimating the Individual Alpha Peak Frequency (iAPF) could improve the neurophysiological specificity of spectral markers, this step was not included in our current preprocessing pipeline as our study was designed to model intra-individual personality phase dynamics and inter-individual trait-group differences rather than perform direct person-wise spectral comparisons. We recognize this as a limitation. Future work will integrate iAPF-based personalized band definitions to further refine the precision and neural specificity of TBR and additional spectral biomarkers.

### 3.4. Classification Model Structures and Feature Selection Techniques

To identify the optimal feature subsets for each personality trait, we applied a classifier-dependent wrapper method for feature selection, tailored separately for SVM and MLP classifiers. This supervised approach, although computationally intensive, maximizes accuracy by testing various combinations of characteristics and selecting those that produce the best results [54]. Using training data, the wrapper algorithm identified the most predictive features for each trait as different traits uniquely influence brain activity. For classification, we used SVM and MLP models, widely recognized in behavioral modeling and stress detection, with a 10-fold cross-validation approach [55]. Feature selection was conducted using Sequential Forward Floating Selection (SFFS), which explores the feature space through greedy hill-climbing combined with backtracking and wrapper-based BestFit evaluation. A 10-fold cross-validation accuracy criterion guided the search for optimal subsets. The procedure employed a local cache size of 1 and terminated after five consecutive non-improving iterations. This process generated distinct electrode–band feature combinations for each personality trait, reflecting the unique neurophysiological patterns associated with traits such as Extraversion, Conscientiousness, and Neuroticism. For SVM, we used polynomial kernel, which is mathematically defined as

(3) $$P\left(a,b\right)={\left(a^{T}b+c\right)}^{deg}$$

where *a* and *b* are defined as input vectors, *c* denotes the penalty parameter constraining the error term, and *deg* specifies the degree of the polynomial. Value of c parameter was 1 for most cases. For the MLP-based classification, an MLP with multilayer transfer functions was employed to map the input features onto the two-class output space. The transfer functions considered include the sigmoid, rectified linear unit (ReLU), and hyperbolic tangent, while the model was trained using the backpropagation algorithm within a supervised learning framework. The training of multilayer perceptrons is carried out by minimizing the squared error between the network’s predicted output and the target values, thereby interpreting the output as an estimate of the class probability, as formulated below:

(4) $$E=\frac{1}{2}{\left(y-f(x)\right)}^{2}$$

where f(x) corresponds to the network’s predicted output, and *y* denotes the true class label of the instance.

## 4. Experimental Results

This study investigates personality dynamics through a multimodal methodology structured into two complementary frameworks. First, we employ neurophysiological data to classify personality traits using machine learning-driven models. Second, we analyze EEG-derived biomarkers to elucidate correlations between personality profiles and neurocognitive health indicators. Prior to these analyses, a validated psychometric assessment was conducted to stratify participants into distinct cohorts based on high and low trait expression, followed by a systematic exploration of interdependencies among these traits.

### 4.1. Personality Analysis

**Figure 4:**
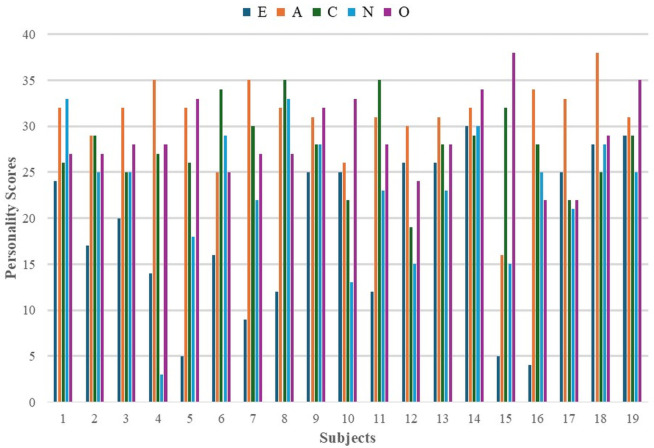
Big Five personality scores for 19 users across Extraversion, Agreeableness, Conscientiousness, Neuroticism, and Openness. Scores were derived from a 50-item questionnaire using trait-specific formulas with reverse scoring as per standard methodology.

Personality traits were assessed using standardized questionnaires, with scores ranging from 0 to 40. Participants were divided into high and low trait groups based on mean scores: 19 for Extraversion, 31 for Agreeableness, 28 for Conscientiousness, 23 for Neuroticism, and 29 for Openness. The scores for the Big Five personality traits were calculated using a standardized scoring method based on a 50-item questionnaire. Each trait score is computed by summing specific items, with some items reverse-scored as per the scoring key. Figure 4 shows the distribution of these traits, where Ex, A, C, N, and O represent Extraversion, Agreeableness, Conscientiousness, Neuroticism, and Openness, respectively. Pearson’s correlation analysis, shown in Table 2, revealed a strong negative correlation between Extraversion and Conscientiousness, suggesting that highly extraverted individuals may be less conscientious (i.e., more flexible). A moderate positive correlation was found between Extraversion and Agreeableness, while Agreeableness was strongly negatively correlated with Openness. Conscientiousness showed a moderate positive correlation with Neuroticism, indicating that emotionally sensitive individuals tend to be more disciplined and mindful. The other traits displayed no significant linear associations, with correlations near zero. Figure 4 shows the personality score distribution of all the participants involved in this study.

### 4.2. Personality Trait Classification Results

#### 4.2.1. Data Annotation

For binary classification, as outlined in Section 3.4, we utilized the SVM and MLP supervised machine learning algorithms with feature labeling based on higher-order versus lower-order personality dimensions. To define the two classes for each personality trait, the mean scores of the personality scale were calculated. This resulted in ten individuals classified as extraverts (Es) and nine as introverts (Is). For A, thirteen individuals exhibited a higher level of A (MA) and six a lower level (LA) of A. Similarly, for C, eleven subjects were classified as having a high level (HC) and eight as having a low level (LC) of C. Regarding the O trait, seven subjects demonstrated a high degree of Openness to Experience (MO), while twelve subjects were classified as having Low Openness (LO). Finally, for Ne, twelve participants were identified as more neurotic (MN) and seven as less neurotic (LN). Several kernel functions were tested for the SVM, with the polynomial kernel selected for this study and the tolerance parameter (C) set to 1 to control errors. A 10-fold cross-validation approach was employed for both SVM and MLP.

#### 4.2.2. Classification Performance

To predict the two classes for each personality trait, five distinct feature groups were derived from the mean band power, calculated using the PSD across 64 EEG electrodes for each trait individually. The performance of SVM and MLP classifiers was evaluated for all personality dimensions, comparing their accuracy, precision, recall, F1 scores, and kappa statistics based on the optimized feature sets. Table 3 presents the selected frequency bands from 64 EEG electrodes for both SVM and MLP across the five personality dimensions.

Table 4 presents the classification results for both classifiers, with the highest accuracy in each case highlighted in bold. We compared the classification accuracy, precision, recall, F1 scores, and kappa statistics for both classifiers across each personality trait. The results are provided for classifying the personality dimensions in all phases of the TSST. Notably, the MLP classifier outperformed the SVM classifier, achieving the highest classification accuracy for Openness at 93.4%, while the SVM classifier also achieved the highest accuracy for Openness at 89.4%. The lowest accuracy was observed for Agreeableness and Conscientiousness when classified using the SVM, with an accuracy of 76.3%, while MLP had the lowest accuracy for Neuroticism at 84.2%.

Additionally, the kappa statistic, which measures the level of agreement or reliability between the predicted values and the ground truth, was analyzed. A kappa value of 0 indicates no agreement, while a value of 1 indicates perfect agreement. The highest kappa value was obtained for Openness, with MLP achieving a kappa of 0.95 and SVM achieving 0.75. The lowest kappa value was observed for Agreeableness (0.31) when classified using SVM.

We also compared classifier performance using precision, recall, and F1 score. The MLP classifier consistently outperformed the SVM classifier across these metrics. The highest precision, recall, and F1 scores (0.93 each) were achieved for Openness using the MLP classifier. In contrast, the lowest precision, recall, and F1 scores for Conscientiousness classification using SVM were 0.82, 0.76, and 0.71, respectively. In conclusion, Openness achieved the best classification results across both classifiers among the Big Five personality traits.

### 4.3. EEG TBR and Personality Trait Correlation

While the relationship between personality traits and cognitive health has been extensively documented in medical and psychological domains, it has received limited attention in computational research. Previous findings suggest that traits such as Openness and Conscientiousness are positively associated with cognitive health and working memory, whereas Neuroticism is often linked to reduced memory performance and slower processing speed [56]. Unlike prior studies that rely primarily on self-reports or behavioral measures, the present work advances this field by directly quantifying these associations through EEG-based brain recordings collected during cognitively demanding tasks. This approach enables a novel correlation analysis that maps personality dimensions to neural markers of cognitive performance, offering a more objective and computationally grounded perspective on personality–cognition interactions. This study explored the potential of theta-to-beta power ratios (TBRs) in the frontal and temporal brain regions to differentiate personality groups across various phases of the Trier Social Stress Test (TSST). Novel insights were uncovered, highlighting significant correlations between TBRs in the frontal and temporal lobes and personality scores. In each TSST phase, associations between personality traits and TBRs from the left frontal (LF), right frontal (RF), left temporal (LT), and right temporal (RT) regions were systematically analyzed. Specifically, TBR was calculated using electrodes located in the frontal region (Fp1, F1, F3, F5, F7, AF7, AF3, Fpz, Fp2, AF8, AF4, Fz, F2, F4, F6, and F8) and the temporal region (T7, TP7, T8, and TP8). Detailed correlation results are presented in Table 5, offering a complete view of the relationship between neural activity and personality traits.

**Figure 5:**
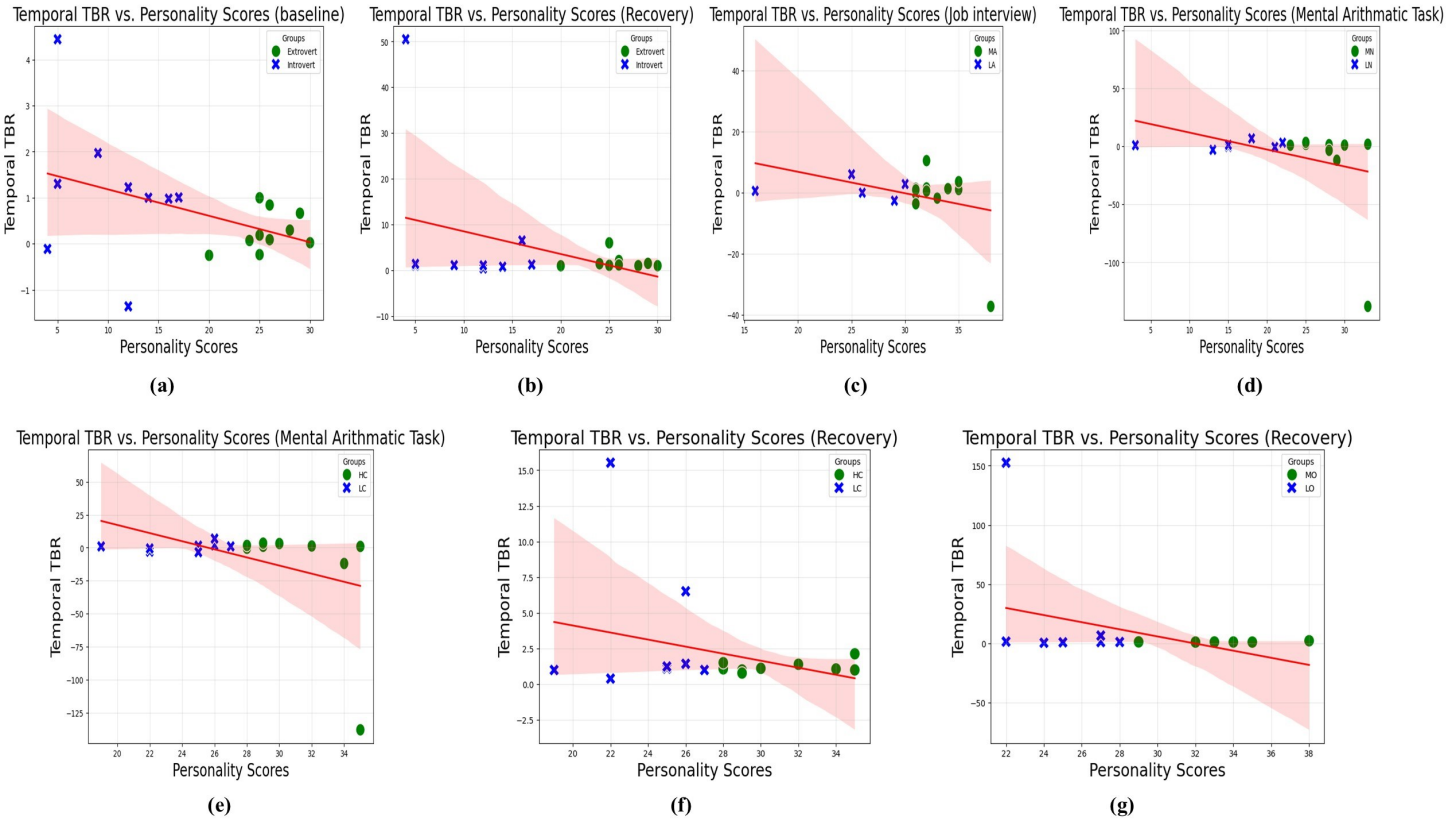
Inverse correlation between personality scores and temporal lobe TBR across TSST phases: (**a**) Extraversion (Baseline), Extrovert vs. Introvert; (**b**) Extraversion (Recovery), Extrovert vs. Introvert group; (**c**) Agreeableness (Job Interview), MA vs. LA group; (**d**) Neuroticism (Mental Arithmetic Task), MN vs. LN group; (**e**) Conscientiousness (Mental Arithmetic Task), HC vs. LC group; (**f**) Conscientiousness (Recovery), HC vs. LC group; (**g**) Openness (Recovery), MO vs. LO group. Higher-order personality group (green circles) vs. lower-order personality group (blue crosses). Red line: correlation trendline; shaded area: 95% confidence interval.

Correlation strength was interpreted using Cohen’s conventional thresholds (small: (r≥±0.10), medium: (r≥±0.30), and large: (r≥±0.50). The method was similar for weak negative and moderate negative correlations. The strong negative or positive correlation threshold was 0.50, which we did not observe [57]. These correlation results are further supported by the trend lines, which illustrate an inverse relationship between personality scores and TBR, as shown in Figure 5.

**Figure 6:**
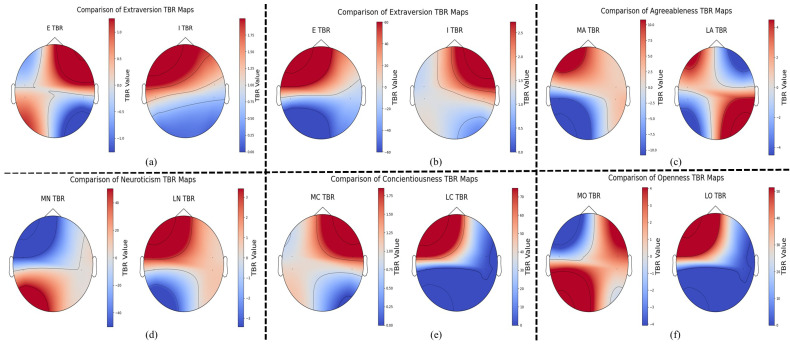
Topographical differences in mean temporal TBR between negatively correlated personality groups. Based on mean temporal TBR, with red indicating heightened TBR and blue indicating lowered TBR. (**a**) Extraversion—Extravert (E) vs. Introvert (I); (**b**) Extraversion under stress—Extravert (E) vs. Introvert (I); (**c**) Agreeableness—MA vs. LA; (**d**) Neuroticism—MN vs. LN; (**e**) Conscientiousness—MC vs. LC; (**f**) Openness—MO vs. LO.Red regions indicate higher TBR values, whereas blue regions indicate lower TBR values.

To gain deeper insights, we compared the theta/beta ratios (TBRs) across different personality trait groups using brain topography (Figure 6). Areas marked in darker red highlight regions of elevated TBR, suggesting greater cognitive decline, whereas darker blue areas reflect reduced TBR, indicative of more stable or enhanced neurocognitive performance. In addition to topographical mapping, we further validated this comparison through correlation analysis, with the results summarized in Figure 5.

Seven moderate negative correlations were identified between temporal TBR and personality scores, as illustrated in Table 5. This section focuses on the most significant correlations, outlined as follows:

#### 4.3.1. Extraversion and Temporal TBR

Extant research has examined the association between Extraversion and cognitive health through multiple physiological modalities, including ECG [58] and EEG recordings [59]. These studies consistently suggest that higher levels of Extraversion act as a protective factor against stress-related deterioration in cognitive functioning. Building on this evidence, the present study advances the field by directly quantifying this relationship through EEG-based neural markers recorded during cognitively demanding tasks, offering a more objective and computationally grounded perspective on the role of Extraversion in cognitive resilience. A moderate negative correlation (−0.41) was observed between Extraversion and temporal TBR during the baseline phase, indicating that extraverted individuals tend to exhibit lower TBR ratios. This suggests that extraverts maintain more stable mental states compared to introverts during resting conditions. Figure 5a illustrates this trend, where an increase in personality scores corresponds to a decrease in TBR, reflecting lower mental fatigue in extraverted participants during baseline conditions.

Following the stress phases, extraverts demonstrated a faster decline in TBR compared to introverts, as evidenced by a significant negative correlation (−0.40). This finding, depicted in Figure 5b, suggests that individuals with higher Extraversion levels recover more rapidly from stress, highlighting their resilience and ability to regain mental stability post-stress in comparison to introverts.

#### 4.3.2. Agreeableness and Temporal TBR

Figure 5c illustrates a negative correlation (−0.34) between A and the temporal TBR during the job interview phase of the TSST. This finding highlights that individuals in the MA group consistently maintain lower TBR levels compared to those in the LA group, underscoring the enhanced resilience to stress and superior cognitive control exhibited by MA individuals in highly stressful environments.

#### 4.3.3. Neuroticism and Temporal TBR

The association between Neuroticism and brain health has also been extensively explored through correlation-based analyses [60]. For N, a negative correlation (−0.34) was observed with temporal TBR during the mental arithmetic task (Figure 5d). High-Neuroticism (HN) individuals exhibited reduced brain activity in the temporal region compared to Low-Neuroticism (LN) individuals during cognitively demanding tasks, highlighting their heightened susceptibility to mental challenges.

#### 4.3.4. Conscientiousness and Temporal TBR

Correlations between brain structure and Conscientiousness have been investigated in prior work [61]. A negative correlation of −0.41 and −0.32 was observed between C and temporal TBR during the mental arithmetic task and recovery period, respectively (Figure 5e,f). The recovery phase patterns indicate that High-Conscientiousness (HC) individuals effectively lower their TBR and restore cognitive function more efficiently compared to LC individuals.

#### 4.3.5. Openness and Temporal TBR

Prior evidence demonstrates a robust positive correlation between Openness to Experience and indicators of positive mental health [62]. Similarly, the MO group demonstrated superior recovery, evidenced by the negative correlation (−0.37) between Openness scores and temporal TBR, as well as the trend illustrated in Figure 5g. Individuals with higher Openness efficiently regained their mental state following stress-inducing phases, such as the mental arithmetic task and job interview, compared to those with lower Openness.

In summary, participants with higher scores in E, C, and O tended to show lower temporal brain rhythms in terms of TBR, indicating better mental stability and quicker recovery from stress-inducing tasks compared to those with lower scores in these traits.

We identified weak correlations between frontal TBR and certain personality traits during specific phases of the TSST. To ensure analytical rigor, we predefined statistical thresholds for interpreting correlation magnitudes based on established guidelines [63]:

- **Weak correlations**: |r|<0.30;
- **Moderate correlations**: 0.30≤|r|<0.50;
- **Strong correlations**: |r|≥0.50 (no instances observed in this study).

These thresholds were applied symmetrically to both positive (r>0) and negative (r<0) associations. While all the correlations—including weaker ones (|r|<0.30)—are fully tabulated in Table 5, only relationships meeting the |r|≥0.30 threshold are emphasized in this section. This conservative approach prioritizes biologically plausible effects while maintaining transparency about marginal associations. These subtle associations may be influenced by individual variability, measurement noise, or confounding factors such as baseline anxiety. For instance, a weak negative correlation between Conscientiousness and frontal TBR was observed both during the baseline and job interview phases. While these findings are consistent with the exploratory scope of our study, further research with larger sample sizes and stronger methodological controls is necessary to validate and clarify these relationships.

## 5. Discussion

**Figure 7:**
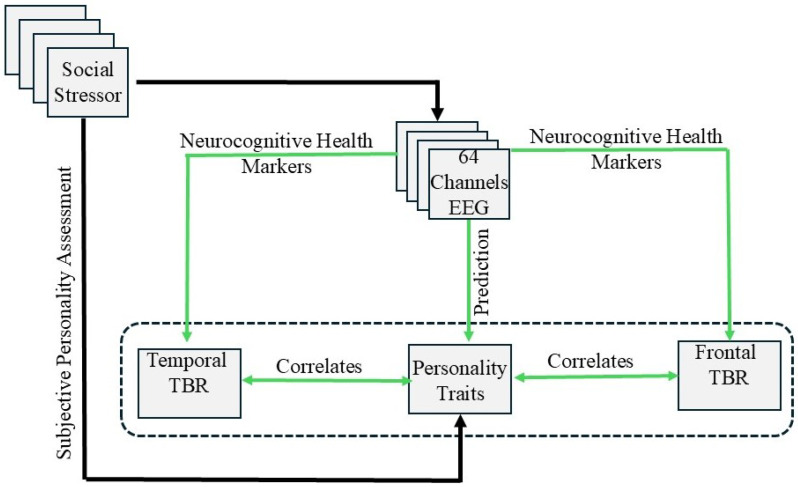
EEG-based computational model for personality identification and neural health markers.

This study introduces an innovative computational framework, pioneering the integration of personality trait identification derived from neurophysiological data acquired during cognitively demanding tasks with a comprehensive evaluation of their impact on cognitive health. Leveraging the TSST paradigm, we systematically analyze dynamic interrelationships between personality dimensions and neural biomarkers across four temporally distinct stress phases—establishing a novel neurocognitive mapping protocol that bridges psychometric profiling, stress reactivity dynamics, and neurological health outcomes. Figure 7 illustrates the computational framework for identifying personality traits and their associations with neural health markers.

The results of personality trait recognition, spectral analysis, and correlation analysis emphasize two important insights. Assessing personality traits by aggregating EEG data across every TSST phase offers a holistic perspective, uncovering consistent neural markers influenced by personality traits across diverse scenarios, as shown in Table 4. Additionally, the distinct brain rhythms observed during various stress phases effectively highlight and characterize personality differences among individuals, as demonstrated in Figure 6 and Table 5.

As delineated in Section 4.2, our neurocomputational framework leveraged the Trier Social Stress Test (TSST) [64], a gold-standard ecologically valid paradigm for probing stress-induced modulations in domain-general cognitive domains (task switching, decision-making, problem-solving, and working memory). Through multiphase EEG recordings (four temporally distinct TSST stages), we decoded the Big Five personality dimensions via machine learning-driven analysis of neurophysiological signatures. Spectral decomposition of neural signals yielded power spectral density (PSD) features across five canonical frequency bands, which were subsequently dichotomized based on established psychometric thresholds. These neural biomarkers served as inputs to optimized support vector machines (SVMs) and multilayer perceptrons (MLPs), achieving robust personality classification through rigorous cross-validation protocols that preserved phase-specific stress dynamics. The MLP demonstrated the highest classification accuracy at 94.7% for A, while the SVM achieved 88.1% accuracy for E.

Finally, Section 4.3 explores how personality traits relate to changes in neural activity during the different stages of the TSST—from the initial baseline to stress-inducing tasks to the recovery period. The correlation results reveal significant patterns between personality traits and brainwave activity. TBR was computed from the frontal and temporal regions of both hemispheres, showing a moderate negative correlation between E and temporal TBR during the baseline and recovery phases. These findings are illustrated in the trendline graphs and brain maps, highlighting the topographies.

At baseline, a moderate negative correlation of −0.41 between E and temporal TBR was found, as shown in Figure 5a. This figure demonstrates that, during the normal condition, the TBR in the temporal region is slightly higher in the I group compared to the E group, with more red areas indicating heightened TBR. During the recovery period, moderate negative correlations of −0.40, −0.32, and −0.37 were observed between the personality scores and temporal TBR for Extraversion (E), Conscientiousness (C), and Openness (O), respectively. These findings are consistent with the trendlines shown in Figure 5b,f,g and the brain maps in Figure 6b,e,f, further supporting the link between personality traits and brainwave activity. Regarding the A trait, a negative correlation of −0.34 was identified between the personality scores and temporal TBR during the job interview phase. For N, a similar negative correlation of −0.34 was observed during the mental arithmetic task. These results are visually represented in the trendlines for A (Figure 5c) and N (Figure 5d), as well as the brain maps for A (Figure 6c) and N (Figure 6d), reinforcing the connection between these traits and brainwave activity. Regarding the relationship between TBR and Extraversion, Figure 5a and Figure 6a reveal consistent patterns. Under normal conditions, extraverts exhibited a slightly more relaxed state and lower fatigue levels compared to introverts, who demonstrated elevated TBR and consequently diminished cognitive health outcomes.

While this study achieved notable classification accuracies for personality traits, greater than state-of-the-art studies [11,12,13,14,16], it faces some limitations. A key observation was the weak or nearly nonexistent correlation between the personality scores and mental responses during certain TSST phases (Figure Table 5). Despite using data from 64 electrodes, the analysis was confined to the frontal and temporal regions. Future research will incorporate multimodal physiological responses for a broader understanding of cognitive health. Additionally, parameters like the theta–alpha ratio (TAR) and coherence [65,66] could be integrated to gain deeper insights into the link between personality traits and cognitive performance. Although individualized alpha peak frequency (iAPF) is recognized as an important and relatively stable neurophysiological marker of cognitive functioning [67], and reductions in peak alpha frequency have been linked to cognitive decline [68], its application is highly context-dependent. The study [69] emphasized the importance of iAPF primarily in sleep, resting-state, and other physiologically stable long-duration recordings [69]. In contrast, the present study utilized short TSST-induced stress epochs characterized by rapid physiological fluctuations and minimal resting-state data, conditions under which iAPF estimation is known to be unreliable and prone to distortion. Moreover, our analysis focuses on group-level theta–beta ratio (TBR) dynamics and personality trait classification derived from canonical EEG frequency bands, a standard practice in acute stress and cognitive-load paradigms. Applying iAPF-based personalized boundaries under these methodological constraints would likely yield unstable spectral estimates and compromise the interpretability of TBR. Therefore, canonical bands were intentionally retained in this pilot framework. Future work using larger samples and extended resting-state baselines will integrate iAPF-based band personalization to enhance spectral specificity.

This study introduces a novel EEG-based computational framework for personality computing, shifting the focus from emotion and stress monitoring to personality trait detection and its influence on behavior across stress phases. Future research will expand the scope of this study by increasing the participant pool to ensure a more diverse and comprehensive understanding of personality traits. It will also incorporate additional physiological signals and health metrics, including factors such as physical activity, sleep patterns, and diet, to provide a holistic view of their interaction with personality traits. By investigating these variables in the context of real-world stressors, the study aims to uncover the intricate relationships between personality traits, cognitive health, and physiological responses. Furthermore, leveraging advanced AI models will be key in developing highly personalized AI-driven healthcare systems that are capable of adapting to individual user profiles with unprecedented accuracy and effectiveness. Our study demonstrates that transparent and resource-efficient pipelines can produce reliable and interpretable results in personality assessment, revealing how brain function varies across individuals and the extent to which personality traits influence these patterns. This approach serves as a baseline for future developments toward more complex and subject-invariant modeling strategies.

In real-world applications, the proposed framework has the potential to be implemented in real-time environments, provided that key factors such as computational cost, model latency, edge-computing feasibility, device compatibility, and user acceptance across different age groups are carefully evaluated. As human-centric AI systems increasingly integrate neurocognitive monitoring and health-related analytics, our work lays important groundwork for personality-informed assessments. By linking EEG-derived neural markers with personality traits, this study opens pathways for developing personalized interventions, adaptive cognitive support, and individualized health recommendations. Nonetheless, responsible deployment will require rigorous attention to ethical considerations, including privacy, consent, data security, and safeguards against misuse of personality profiling. Previous research has shown that, in women, both personality-related stress responses and EEG spectral patterns can be influenced by menstrual cycle phase due to hormonal fluctuations. In the present study, our primary objective was to identify personality-linked neural patterns and subsequently examine how these personality traits relate to neurocognitive health biomarkers such as TBR. Because TBR-based neurocognitive markers and personality classifications are fundamentally gender-independent constructs, menstrual cycle tracking was not integrated into the initial experimental design. However, given that the majority of our participants were women, we acknowledge that fluctuations in ovarian hormones across the menstrual cycle may modulate stress reactivity, personality expression, and EEG frequency characteristics. This represents an important methodological limitation. In future work, we will incorporate systematic menstrual cycle monitoring and phase-specific scheduling to minimize hormonal confounds and thereby strengthen the precision and physiological interpretability of personality–stress–EEG relationships.

## 6. Conclusions

This study introduces a novel framework for implicit personality trait recognition via EEG signals collected from 21 participants using 64 Ag/AgCl active scalp electrodes during a TSST-based task, alongside explicitly acquired Big Five personality scores. From the preprocessed EEG data, PSD bands were extracted and selected to evaluate personality classification performance using two machine learning models, SVM and MLP. Our approach achieved superior classification accuracy compared to several state-of-the-art methods.

Beyond personality recognition, we explored the relationship between personality traits and neurocognitive health using TBR as a predictor of cognitive health. We investigated the influence of personality traits on cognitive performance by examining correlations between the personality scores and frontal and temporal TBR during each TSST phase.

This study advances research on the relationship between personality, stress, and cognitive health. It not only identifies personality differences but also explores their impact on individual cognitive well-being. Our findings establish important benchmarks in this area. Future research will integrate diverse psychophysiological data, employ longitudinal studies, examine more naturalistic settings and stressors, explore clinical applications, and leverage advanced AI techniques to deepen understanding and generate new insights. Although our dataset includes 19 valid participants, we acknowledge that this small sample limits statistical power and generalizability. Therefore, our findings should be considered preliminary. Future studies will use larger and more diverse samples to validate and strengthen the model’s robustness.

## Figures and Tables

**Table 1:** Comparison of state-of-the-art studies on personality trait recognition using physiological signals.

Ref.	Year	Sensors	Model	Setting	Methodology	Key Contributions	Limitations
[11]	2020	EEG, PPG, GSR	Big Five	Public speaking	KNN classification using time–frequency EEG features	Differentiated personality traits using physiological responses	Short recording duration; limited EEG channels; no personality–health analysis
[12]	2017	EEG	BFI + ERQ	Emotional movie clips	SVM-based classification on EEG emotional responses	Showed influence of emotional context on personality-related neural activity	Limited electrodes; lacks direct personality–emotion modeling
[13]	2020	EEG	BFI	28 emotional video clips	EEG analysis combined with self-reports	Estimated Big Five traits from neural patterns	Correlation-only; unclear role of neural dynamics
[14]	2020	EEG	BFI	Emotional video stimuli	SVM classification into high/low personality scales	Used ASCERTAIN dataset for EEG-based personality recognition	Small dataset; only 8 EEG channels; trait-specific effects unclear
[15]	2023	EMG, GSR, ECG	Big Five	Online teaching	SVM per-trait from physiological signals	Proposed teacher personality evaluation using biosignals	No EEG; no neurocognitive link
[16]	2016	EEG, ECG, GSR	Big Five	Affective video trials	Naive Bayes and SVM with uni-/multimodal fusion	Explored personality–affect statistical relations	Weak correlations; emotional stimuli only
Our Study	–	EEG	Big Five Scale	Social stress (TSST)	PSD features + SVM; cognitive biomarkers from frontal/temporal regions	Links personality traits with neurocognitive stress response using 64-channel EEG	Exploratory sample (21 subjects); needs larger validation

**Table 2:** Pearson correlation matrix of personality traits.

Traits	Ex	A	C	N	O
Ex	1	0.22	−0.46 **	0.05	−0.10
A	0.22	1	−0.18	−0.08	−0.46 **
C	−0.46 **	−0.18	1	0.37 *	0.02
N	0.05	−0.08	0.37 *	1	0.10
O	−0.10	−0.46 **	0.02	0.10	1

**Note:** * *p* < 0.05, ** *p* < 0.01 (two-tailed Pearson correlation).

**Table 3:** Selected features for SVM and MLP classifiers by personality trait, band, and electrode.

SVM Classifier			MLP Classifier	
Trait	Band	Electrodes	Band	Electrodes
**Ex**	Δ	Iz, O2	Δ	C1, O2
	Θ	–	Θ	Lz, O2
	α	T7, F8, Cz	α	P5, PO4
	β	P8	β	CPz, P6
	γ	P6	γ	Cz
**A**	Δ	P9	Δ	Lz, POz
	θ	O1	θ	C1, PO3
	α	P9, T8	α	T8
	β	–	β	–
	γ	O1	γ	O2
**C**	Δ	AF4, T8, CP6	Δ	F3, T7
	θ	Lz, Fz	θ	–
	α	FP1, PO7,	α	CPz
	β	FP1, FC5, Lz, CPz, FPz	β	C2
	γ	FT7, PO3, C6, PO4	γ	Lz
**O**	Δ	F1, CP1, F2	Δ	P3, PO7, P4
	θ	–	θ	T8
	α	P1, T8	α	CP5, CP1
	β	–	β	FC3, F2, F6
	γ	F5, F8, CP6, O2	γ	FC1, O2
**N**	Δ	Cp5, P7, Lz, Pz, FT8, Cz, PO8, O2	Δ	C4
	θ	Fc5	θ	P7, FC2
	α	FT7, PO7, FPz Po8, F8	α	–
	β	F1, F3, FT7, Pz, CP4	β	AF3, C6
	γ	FP1, C6, PO4	γ	F5, Lz

**Table 4:** Performance comparison of SVM and MLP models for personality trait identification during overall TSST.

Trait	Model	Accuracy	Precision	Recall	F-Measure	Ka
Ex	SVM	81.5	0.80	0.80	0.81	0.62
	MLP	88.1	0.88	0.88	0.88	0.76
A	SVM	76.3	0.82	0.76	0.71	0.31
	MLP	94.7	0.95	0.94	0.94	0.96
N	SVM	81.5	0.82	0.81	0.80	0.57
	MLP	84.2	0.84	0.84	0.84	0.66
C	SVM	76.3	0.83	0.76	0.73	0.47
	MLP	81.5	0.81	0.82	0.81	0.62
O	SVM	89.4	0.91	0.89	0.89	0.75
	MLP	93.4	0.93	0.93	0.93	0.95

**Table 5:** TBR and personality trait correlations across TSST phases.

TSST Phases	Region	Ex	A	N	C	O
**Baseline**	Frontal TBR	0.03	0.05	0.05	−0.14	0.20
	Temporal TBR	**−0.41**	−0.01	−0.18	−0.04	−0.21
**Arithmetic Task**	Frontal TBR	0.19	0.01	−0.29	−0.22	−0.01
	Temporal TBR	−0.21	−0.03	**−0.34**	**−0.41**	0.03
**Job Interview**	Frontal TBR	0.22	0.22	0.25	−0.13	0.06
	Temporal TBR	−0.22	**−0.34**	−0.09	0.16	0.01
**Recovery**	Frontal TBR	−0.17	−0.20	−0.05	−0.14	−0.25
	Temporal TBR	**−0.40**	0.11	0.06	**−0.32**	**−0.37**

**Note:** Bold values indicate relatively stronger correlations (higher absolute Pearson *r* values) between TBR and personality traits across TSST phases.

## Data Availability

The annotated EEG dataset and personality labels have been publicly shared for reproducibility and useful research. GitHub repository for the EEG+personality dataset: https://github.com/Majid-38/EEG-Dataset-for-Psychosocial-Stress-TSST-with-Personality-Labels (accessed on 7 May 2025).

## References

[1] McAdams D.P., Olson B.D. (2010). Personality development: Continuity and change over the life course. Annu. Rev. Psychol..

[2] Digman J.M. (1989). Five robust trait dimensions: Development, stability, and utility. J. Personal..

[3] Riaz M., Majid M., Mir J. (2024). High dynamic range multimedia: Better affective agent for human emotional experience. Multimed. Tools Appl..

[4] Ji Y., Wu W., Zheng H., Hu Y., Chen X., He L. (2023). Is chatgpt a good personality recognizer? a preliminary study. arXiv.

[5] De Smet S., Razza L.B., Pulopulos M.M., De Raedt R., Baeken C., Brunoni A.R., Vanderhasselt M.A. (2024). Stress priming transcranial direct current stimulation (tDCS) enhances updating of emotional content in working memory. Brain Stimul..

[6] Villada C., Hidalgo V., Almela M., Salvador A. (2016). Individual differences in the psychobiological response to psychosocial stress (Trier Social Stress Test): The relevance of trait anxiety and coping styles. Stress Health.

[7] Terracciano A., Stephan Y., Luchetti M., Albanese E., Sutin A.R. (2017). Personality traits and risk of cognitive impairment and dementia. J. Psychiatr. Res..

[8] Luchetti M., Terracciano A., Stephan Y., Aschwanden D., Sutin A.R. (2021). Personality traits and memory: A multilevel analysis across 27 countries from the survey of health, ageing and retirement in Europe. Psychol. Sci..

[9] Azami H., Zrenner C., Brooks H., Zomorrodi R., Blumberger D.M., Fischer C.E., Flint A., Herrmann N., Kumar S., Lanctôt K. (2023). Beta to theta power ratio in EEG periodic components as a potential biomarker in mild cognitive impairment and Alzheimer’s dementia. Alzheimer’s Res. Ther..

[10] Vatsal R., Mishra S., Thareja R., Chakrabarty M., Sharma O., Shukla J. (2024). An analysis of physiological and psychological responses in virtual reality and flat screen gaming. IEEE Trans. Affect. Comput..

[11] Butt A.R., Arsalan A., Majid M. (2020). Multimodal personality trait recognition using wearable sensors in response to public speaking. IEEE Sens. J..

[12] Zhao G., Ge Y., Shen B., Wei X., Wang H. (2017). Emotion analysis for personality inference from EEG signals. IEEE Trans. Affect. Comput..

[13] Li W., Hu X., Long X., Tang L., Chen J., Wang F., Zhang D. (2020). EEG responses to emotional videos can quantitatively predict big-five personality traits. Neurocomputing.

[14] Annisa F.Q., Supriyanto E., Taheri S. Personality dimensions classification with EEG analysis using support vector machine. Proceedings of the 2020 3rd International Seminar on Research of Information Technology and Intelligent Systems (ISRITI).

[15] Singh J., Arya R. (2023). Examining the relationship of personality traits with online teaching using emotive responses and physiological signals. Educ. Inf. Technol..

[16] Subramanian R., Wache J., Abadi M.K., Vieriu R.L., Winkler S., Sebe N. (2016). ASCERTAIN: Emotion and personality recognition using commercial sensors. IEEE Trans. Affect. Comput..

[17] Leekha M., Khan S.N., Srinivas H., Shah R.R., Shukla J. (2024). VyaktitvaNirdharan: Multimodal Assessment of Personality and Trait Emotional Intelligence. IEEE Trans. Affect. Comput..

[18] Hu F., He K., Wang C., Zheng Q., Zhou B., Li G., Sun Y. (2025). STRFLNet: Spatio-Temporal Representation Fusion Learning Network for EEG-Based Emotion Recognition. IEEE Trans. Affect. Comput..

[19] Fan C., Wang J., Huang W., Yang X., Pei G., Li T., Lv Z. (2024). Light-weight residual convolution-based capsule network for EEG emotion recognition. Adv. Eng. Inform..

[20] Goldberg L.R. (1999). A broad-bandwidth, public domain, personality inventory measuring the lower-level facets of several five-factor models. Personality Psychology in Europe.

[21] John O.P., Naumann L.P., Soto C.J. (2008). Paradigm shift to the integrative big five trait taxonomy. Handbook of Personality: Theory and Research.

[22] Peters H., Matz S.C. (2024). Large language models can infer psychological dispositions of social media users. PNAS Nexus.

[23] Kim Y. (2024). Personality of organizational social media accounts and its relationship with characteristics of their photos: Analyses of startups’ Instagram photos. BMC Psychol..

[24] Mark J.A., Curtin A., Kraft A.E., Ziegler M.D., Ayaz H. (2024). Mental workload assessment by monitoring brain, heart, and eye with six biomedical modalities during six cognitive tasks. Front. Neuroergonomics.

[25] Moser M.K., Resch B., Ehrhart M. (2023). An individual-oriented algorithm for stress detection in wearable sensor measurements. IEEE Sens. J..

[26] Zhou Y., Huang S., Xu Z., Wang P., Wu X., Zhang D. (2021). Cognitive workload recognition using EEG signals and machine learning: A review. IEEE Trans. Cogn. Dev. Syst..

[27] Tamantini C., Cristofanelli M.L., Fracasso F., Umbrico A., Cortellessa G., Orlandini A., Cordella F. (2025). Physiological Sensor Technologies in Workload Estimation: A Review. IEEE Sens. J..

[28] Angkan P., Behinaein B., Mahmud Z., Bhatti A., Rodenburg D., Hungler P., Etemad A. (2024). Multimodal Brain–Computer Interface for In-Vehicle Driver Cognitive Load Measurement: Dataset and Baselines. IEEE Trans. Intell. Transp. Syst..

[29] Wang Q., Yang S., Liu M., Cao Z., Ma Q. (2014). An eye-tracking study of website complexity from cognitive load perspective. Decis. Support Syst..

[30] Seixas F.L., Zadrozny B., Laks J., Conci A., Saade D.C.M. (2014). A Bayesian network decision model for supporting the diagnosis of dementia, Alzheimer’s disease and mild cognitive impairment. Comput. Biol. Med..

[31] Stuiver A., Mulder B. (2014). Cardiovascular state changes in simulated work environments. Front. Neurosci..

[32] Narayana V.V., Kodali P. (2025). Enhanced epilepsy sensitivity and detection rate with improved specificity by integration of modified lstm networks. IEEE Sens. J..

[33] Zubair M., Belykh M.V., Naik M.U.K., Gouher M.F.M., Vishwakarma S., Ahamed S.R., Kongara R. (2021). Detection of epileptic seizures from EEG signals by combining dimensionality reduction algorithms with machine learning models. IEEE Sens. J..

[34] Khare S.K., Bajaj V., Acharya U.R. (2021). PDCNNet: An automatic framework for the detection of Parkinson’s disease using EEG signals. IEEE Sens. J..

[35] Putman P., Verkuil B., Arias-Garcia E., Pantazi I., van Schie C. (2014). EEG theta/beta ratio as a potential biomarker for attentional control and resilience against deleterious effects of stress on attention. Cogn. Affect. Behav. Neurosci..

[36] Wen T.Y., Aris S.M. (2020). Electroencephalogram (EEG) stress analysis on alpha/beta ratio and theta/beta ratio. Indones. J. Electr. Eng. Comput. Sci..

[37] Giannakakis G., Grigoriadis D., Giannakaki K., Simantiraki O., Roniotis A., Tsiknakis M. (2019). Review on psychological stress detection using biosignals. IEEE Trans. Affect. Comput..

[38] Seo S., Gil Y., Lee J. The relation between affective style of stressor on EEG asymmetry and stress scale during multimodal task. Proceedings of the 2008 Third International Conference on Convergence and Hybrid Information Technology.

[39] Li P., Yokoyama M., Okamoto D., Nakatani H., Yagi T. (2024). Resting-state EEG features modulated by depressive state in healthy individuals: Insights from theta PSD, theta-beta ratio, frontal-parietal PLV, and sLORETA. Front. Hum. Neurosci..

[40] Chang J., Choi Y. (2023). Depression diagnosis based on electroencephalography power ratios. Brain Behav..

[41] Habibovic I., Almisreb A., Hodzic M., Turaev S., Cantelli-Forti A. (2025). Machine Learning Algorithms and Sensor Technologies for Aging-in-Place Applications: A Review. IEEE Sens. J..

[42] Wertz J., Israel S., Arseneault L., Belsky D.W., Bourassa K.J., Harrington H., Houts R., Poulton R., Richmond-Rakerd L.S., Røysamb E. (2021). Vital personality scores and healthy aging: Life-course associations and familial transmission. Soc. Sci. Med..

[43] Riaz M., Gravina R. (2024). Wearable Sensor Systems to Detect Biomarkers of Personality Traits for Healthy Aging: A Review. IEEE Sens. J..

[44] Riaz M., Gravina R. Anxiety and EEG Frontal Theta-Beta Ratio Relationship Analysis Across Personality Traits During HDR Affective Videos Experience. Proceedings of the ICT4AWE.

[45] Canli T. (2004). Functional brain mapping of extraversion and neuroticism: Learning from individual differences in emotion processing. J. Personal..

[46] Miranda-Correa J.A., Abadi M.K., Sebe N., Patras I. (2018). Amigos: A dataset for affect, personality and mood research on individuals and groups. IEEE Trans. Affect. Comput..

[47] Uddin M.Z., Hassan M.M. (2018). Activity recognition for cognitive assistance using body sensors data and deep convolutional neural network. IEEE Sens. J..

[48] Greco A., Valenza G., Lazaro J., Garzon-Rey J.M., Aguilo J., de la Cámara C., Bailón R., Scilingo E.P. (2021). Acute stress state classification based on electrodermal activity modeling. IEEE Trans. Affect. Comput..

[49] Richer R., Koch V., Abel L., Hauck F., Kurz M., Ringgold V., Müller V., Küderle A., Schindler-Gmelch L., Eskofier B.M. (2024). Machine learning-based detection of acute psychosocial stress from body posture and movements. Sci. Rep..

[50] Rodrigues S., Paiva J.S., Dias D., Aleixo M., Filipe R.M., Cunha J.P.S. (2018). Cognitive impact and psychophysiological effects of stress using a biomonitoring platform. Int. J. Environ. Res. Public Health.

[51] Alarcón-Segovia L.C., Morel R., Spies R., Rintoul I. (2024). The challenging scalp–electrode interface and the evolution of materials and electrode integrated icts for electroencephalography. Surf. Rev. Lett..

[52] Daadaa Y. (2024). EmotionNet: Dissecting Stress and Anxiety Through EEG-based Deep Learning Approaches. Int. J. Adv. Comput. Sci. Appl..

[53] Jenke R., Peer A., Buss M. (2014). Feature extraction and selection for emotion recognition from EEG. IEEE Trans. Affect. Comput..

[54] Maseno E.M., Wang Z. (2024). Hybrid wrapper feature selection method based on genetic algorithm and extreme learning machine for intrusion detection. J. Big Data.

[55] Kyrou M., Kompatsiaris I., Petrantonakis P.C. (2025). Deep Learning Approaches for Stress Detection: A Survey. IEEE Trans. Affect. Comput..

[56] Sutin A.R., Luchetti M., Aschwanden D., Sesker A.A., Zhu X., Stephan Y., Terracciano A. (2023). Five-factor model personality domains and facets associated with markers of cognitive health. J. Individ. Differ..

[57] Brydges C.R. (2019). Effect size guidelines, sample size calculations, and statistical power in gerontology. Innov. Aging.

[58] Brouwer A.M., Van Schaik M.G., Korteling J., van Erp J.B., Toet A. (2014). Neuroticism, extraversion, conscientiousness and stress: Physiological correlates. IEEE Trans. Affect. Comput..

[59] Rogala J., Dreszer J., Malinowska U., Waligóra M., Pluta A., Antonova I., Wróbel A. (2021). Stronger connectivity and higher extraversion protect against stress-related deterioration of cognitive functions. Sci. Rep..

[60] Ueda I., Kakeda S., Watanabe K., Sugimoto K., Igata N., Moriya J., Takemoto K., Katsuki A., Yoshimura R., Abe O. (2018). Brain structural connectivity and neuroticism in healthy adults. Sci. Rep..

[61] Wang S., Zhao Y., Li J., Wang X., Luo K., Gong Q. (2019). Brain structure links trait conscientiousness to academic performance. Sci. Rep..

[62] Lamers S.M., Westerhof G.J., Kovács V., Bohlmeijer E.T. (2012). Differential relationships in the association of the Big Five personality traits with positive mental health and psychopathology. J. Res. Personal..

[63] Chuan C.L., Penyelidikan J. (2006). Sample size estimation using Krejcie and Morgan and Cohen statistical power analysis: A comparison. J. Penyelid. IPBL.

[64] Allen A.P., Kennedy P.J., Cryan J.F., Dinan T.G., Clarke G. (2014). Biological and psychological markers of stress in humans: Focus on the Trier Social Stress Test. Neurosci. Biobehav. Rev..

[65] Bian Z., Li Q., Wang L., Lu C., Yin S., Li X. (2014). Relative power and coherence of EEG series are related to amnestic mild cognitive impairment in diabetes. Front. Aging Neurosci..

[66] Kaushik P., Shrivastava P.K. (2024). Remediation of Learning Difficulty Utilizing School-Based Cognitive Behavioral Intervention Measured by EEG Theta-Alpha and Theta-Beta Ratio During Resting and Cognitive Task Performance Conditions. Clin. EEG and Neurosci..

[67] Grandy T.H., Werkle-Bergner M., Chicherio C., Schmiedek F., Lövdén M., Lindenberger U. (2013). Peak individual alpha frequency qualifies as a stable neurophysiological trait marker in healthy younger and older adults. Psychophysiology.

[68] Lavy Y., Dwolatzky T., Kaplan Z., Guez J., Todder D. (2019). Neurofeedback improves memory and peak alpha frequency in individuals with mild cognitive impairment. Appl. Psychophysiol. Biofeedback.

[69] Chapman R., Najima S., Tylinski Sant’Ana T., Lee C.C.K., Filice F., Babineau J., Mollayeva T. (2025). Sex differences in electrical activity of the brain during sleep: A systematic review of electroencephalographic findings across the human lifespan. BioMed. Eng. OnLine.

